# Measurement of the Radial Nerve Danger Zone in Filipino Adults: A Cadaveric Study

**DOI:** 10.5704/MOJ.2111.007

**Published:** 2021-11

**Authors:** DA Rubio, A Pacheco, A Abrilla

**Affiliations:** 1 Philippine General Hospital, University of the Philippines Manila, Manila, Philippines; 2 College of Medicine, University of the Philippines Manila, Manila, Philippines

**Keywords:** cadaver, danger zone, Filipino, humerus, radial nerve

## Abstract

**Introduction:**

The radial nerve danger zone (RNDZ) is an important anatomic consideration to anticipate or prevent injury in trauma assessment or surgical fixation. No published estimate currently exists for Filipinos. In this study, we sought to provide a local estimate and explore potential predictors of this anatomic region in Filipino adult cadavers.

**Materials and methods:**

Posterior dissection to expose and measure the radial nerve, from the lateral epicondyle to the lateral intermuscular septum, was performed in 60 upper limbs from 30 formalin-preserved cadavers in the laboratory of the Department of Anatomy, College of Medicine, University of the Philippines Manila. Univariate and multivariate linear regression modelling was performed with RNDZ as the dependent variable and age, sex, height and humeral length as potential independent variables individually and in combinations.

**Results:**

The mean radial nerve length from the lateral epicondyle to the lateral intermuscular septum was estimated at 10.6 cm (95% confidence interval: 10.3 cm, 10.9cm). Height and humeral length were statistically significant univariate predictors in female cadavers, while only height was significant in male cadavers. In addition, all multivariate regression models were statistically significant and accounted for more than 57% of the variability in female RNDZ estimates. In comparison, only models that included height and age were statistically significant predictors of RNDZ and accounted for at most 22% of the variability of the estimate in males.

**Conclusion:**

The estimated length of the radial nerve danger zone generated in this study should be strongly considered over other published estimates in surgical fixation procedures performed in adult Filipinos.

## Introduction

The radial nerve provides substantial motor and sensory function to the upper extremity. Arising from the posterior cord of the brachial plexus, this nerve crosses medial to lateral obliquely, along with accompanying vessels, over the posterior surface of the humeral spiral groove. It then penetrates the lateral intramuscular septum near the junction of the middle and distal thirds of the humerus^1^. Disruption of this structure often leads to clinically relevant motor consequences, such as the inability to extend the elbow, wrist and fingers and paraesthesia along its sensory distribution. Such palsy often complicates conditions or interventions involving the humerus, which is often attributed to the intimate association between the course of the nerve and the long bone in question^2^.

Approximately 5% to 30% of such injuries are iatrogenic, secondary to humeral surgical fixation instruments (such as surgical blades, Kirschner wires, plates and screws, and external fixators) and procedures (such as fracture manipulation, limb traction and even improper positioning on the operating table)^3-6^. Since this aetiology or mechanism is preventable, at least theoretically, orthopaedic surgeons having a comprehensive understanding of the anatomical relations of the radial nerve is a must. Furthermore, in order to describe further the anatomical relations of the radial nerve along its tract in the distal humerus, its direct visualisation is necessary; this, in turn, will help design measures to avoid injuring the nerve during surgical management.

A component of this practical knowledge is the so-called “danger zone” of the radial nerve, a region in the distal humerus where the nerve runs posteriorly from lateral epicondyle to the lateral intermuscular septum and where surgical fixation must be avoided, lest the risk for radial nerve palsy is substantially heightened^3,7^. Various estimates for this region exist in the literature, with some studies showing large variability in the course of the nerve within the 6cm to 16cm range^8,9^. Nevertheless, the applicability of these estimates in the Filipino anatomy remains yet to be established.

This study thus aimed to generate an estimate of the radial nerve danger zone (RNDZ) in adult Filipino cadavers.

## Materials and Methods

This observational cross-sectional study was reviewed by the University of the Philippines Manila Research Ethics Board (under protocol code 2020-583-EX) in line with the 2017 Philippine National Ethical Guidelines for Health and Health-Related Research^10^. The procedures performed herein followed the technical and ethical standards of the institutional affiliations of all investigators. All formalin-preserved cadavers from the teaching laboratory of the Department of Anatomy, College of Medicine, the University of the Philippines Manila at the time of study implementation were considered. The age at death and anatomic sex of each cadaver were also obtained from anonymised institutional records. Upper limbs with gross apparent upper extremity deformities, if any, were excluded.

**Fig 1:**
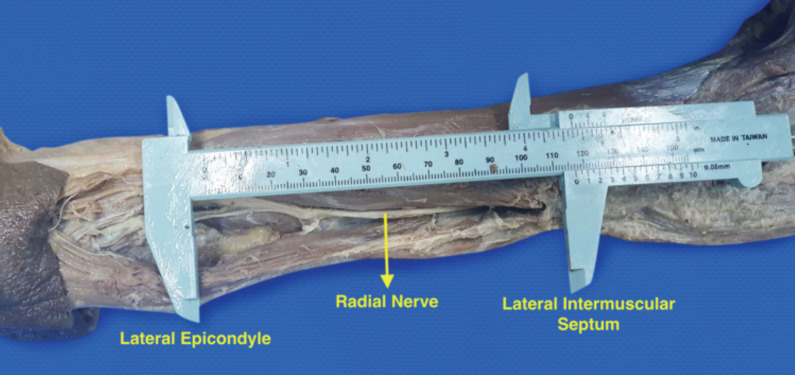
Measurement orientation of radial nerve danger zone from the tip of the lateral epicondyle to the lateral intermuscular septum.

After measuring and recording the height of each cadaver and the lengths of their humeri with a tape measure, a posterior dissection of the distal humeral area was performed to expose the lateral epicondyle. The radial nerve was then located with its course followed up to the lateral intermuscular septum. In the process, the said nerve was not manipulated to maintain its preserved anatomic relations. The RNDZ – the length of the exposed radial nerve in the posterior arm from the lateral epicondyle to its insertion to the lateral intermuscular septum – was measured and recorded in each upper limb through a Vernier calliper (Fig. 1).

Mean, standard deviation (SD) and 95% confidence interval (CI) were utilised to generate summary estimates of cadaver height, humeral length and the danger zone, with stratification by recorded anatomic sex. To assess the statistically significant difference at p < 0.0500, unequal variances (Welch) t-test was performed to compare the summary measures in terms of upper limb laterality and recorded anatomic sex. The absence of statistically significant difference (set at p > 0.0500) between left and right humeral length and danger zone was used to further analyse the measurements without regard to laterality or sex. To determine whether any or a combination of the other measured variables predict the danger zone, linear regression analyses were also performed. Stata/IC version 14.2^11^ was utilised for these analytical purposes.

## Results

A total of 30 cadavers (12 females and 18 males), each with two intact upper limbs, were included in the study (Table I). The mean (SD) age at death of the cadavers were 52.4 (16.8) years, ranging between 24 and 83 years, and the mean (SD) height was 157.4 (9.4) cm. The mean (SD) length of both the left and right humeri was estimated at 30.2 (2.4) cm (difference not statistically significant, p = 0.1136). In comparison, the mean (SD) left, and right RNDZ (the length of the exposed nerve from the lateral epicondyle to the lateral intermuscular septum insertion) were measured at 10.6 (1.1) cm and 10.6 (1.2) cm (difference not statistically significant, p = 0.9399), respectively.

**Table I:** Measured characteristics of the cadavers included in the study

Variable	Total (n = 30)	Females (n = 12)	Males (n = 18)	p-value
Age in years, mean (SD)	52.4 (16.8)	56.6 (19.0)	49.6 (15.0)	0.2937
Height in cm, mean (SD)	157.4 (9.4)	150.1 (7.8)	162.2 (7.0)	0.0002*
Humeral length in cm, mean (SD)				
Left (30 limbs)	30.2 (2.4)	29.0 (2.4)	31.1 (2.0)	0.0214*
Right (30 limbs)	30.2 (2.4)	28.9 (2.3)	31.0 (2.0)	0.0150*
Pooled (60 limbs)	30.2 (2.4)	28.9 (2.3)	31.1 (2.0)	0.0006*
Danger zone in cm, mean (SD)				
Left (30 limbs)	10.6 (1.1)	10.4 (0.9)	10.8 (1.3)	0.3477
Right (30 limbs)	10.6 (1.2)	10.4 (0.9)	10.8 (1.3)	0.3302
Pooled (60 limbs)	10.6 (1.1)	10.4 (0.9)	10.8 (1.3)	0.1669
Danger zone as percentage (%) of humeral length, mean (SD)				
Left (30 limbs)	35.3 (3.9)	36.1 (3.4)	34.7 (4.2)	0.3474
Right (30 limbs)	35.3 (3.9)	36.1 (3.1)	34.9 (4.2)	0.3750
Pooled (60 limbs)	35.3 (3.9)	36.1 (3.2)	34.8 (4.2)	0.1865

*Significant at p < 0.05.

*Abbreviation* - cm: centimetre, n: number of cadavers, SD: standard deviation

**Fig 2:**
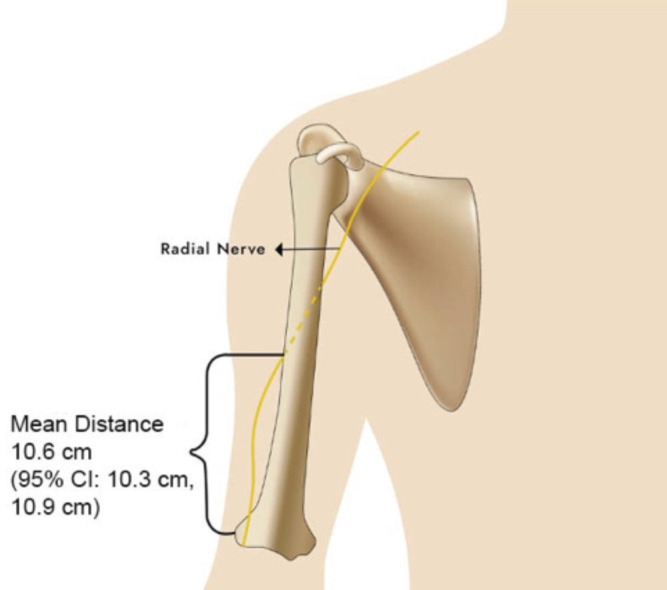
Graphic representation of the estimated mean radial nerve danger zone (RNDZ), the distance from the lateral epicondyle to the lateral intermuscular septum at the spiral groove, from the studied cadavers. (CI: confidence interval).

While the cadavers compared by sex at birth revealed statistically similar mean age (p = 0.2938), the male cadavers were taller (mean [95% CI] difference in height: 12.1cm [6.4cm, 17.9cm], p = 0.0002) and had longer left (2.1cm [0.3 cm, 3.9 cm], p = 0.0214) and right (2.2cm [0.5cm, 3.9cm], p = 0.0150) humeri than female cadavers. Nevertheless, the left (p = 0.3477) and right (p = 0.3302) RNDZ did not statistically differ by this stratification, as were the left and right humeral lengths (p > 0.0500 in all comparisons). As the danger zone did not statistically differ by sex at birth and laterality, the pooled mean (95% CI) estimate for this parameter, using the entire sample and disregarding laterality (60 upper limbs in total), is 10.6cm (10.3cm, 10.9cm), or 34.8% (33.4%, 36.2%) of the humeral length (Fig. 2).

With the data collected for age at death, sex at birth, height and humeral length in each cadaver as potential predictors, univariate linear regression analyses with the RNDZ as the dependent variable were performed. Among females (n = 12, 24 upper limbs), height and humeral length positively correlated with the RNDZ and were revealed to account for 57.74% and 17.83%, respectively, of the variation in this measure (p < 0.0500 in both cases, Table II). On the other hand, height was the only statistically significant predictor of this variable in males (n = 18, 36 upper limbs), accounting for only 13.57% of the observed variation. When the cadavers were analysed regardless of sex, height (R^2^ = 22.68%) and humeral length (R^2^ = 9.54%) emerged as statistically significant predictors of the dependent variable.

**Table II:** Univariate linear regression analysis results for radial nerve danger zone (RNDZ) in female, male and the whole sample of cadavers

Potential Predictor	Coefficient (SE)	p-value	R	R^2^	Intercept (SE)
Female Subset of Sample					
Age	-0.0059 (0.0010)	0.5621	0.1245	0.0155	10.7279 (0.5926)
Equation: RNDZ = 10.7279 – 0.0059(A)					
Height	0.0875 (0.0160)	0.0000*	0.7599	0.5774	-2.7321 (2.3976)
Equation: RNDZ = 0.0875(H) – 2.7321					
Humeral length	0.1584 (0.0725)	0.0398*	0.4223	0.1783	5.8148 (2.1029)
Equation: RNDZ = 0.1584(L) + 5.8148					
Male Subset of Sample					
Age	-0.0144 (0.0145)	0.3285	0.1676	0.0281	11.4976 (0.7507)
Equation: RNDZ = 11.4976 – 0.0144(A)					
Height in cm	0.0677 (0.0293)	0.0271*	0.3684	0.1357	-0.1988 (4.7576)
Equation: RNDZ = 0.0677(H) – 0.1988					
Humeral length in cm	0.1225 (0.1062)	0.2568	0.1942	0.0377	5.8148 (2.1029)
Equation: RNDZ = 0.1225(L) + 5.8148					
Full Aggregate of Sample					
Age	-0.0120 (0.0088)	0.1793	0.1676	0.1758	11.2574 (0.4851)
Equation: RNDZ = 11.2574 – 0.0120(A)					
Female sex	-0.3875 (0.2975)	0.1979	0.1685	0.0284	10.7833 (0.1882)
Equation: RNDZ = 10.7833 – 0.3875(F)					
Male sex	0.3875 (0.2975)	0.1979	0.1685	0.0284	10.3958 (0.2305)
Equation: RNDZ = 0.3875(M) + 10.3958					
Height in cm	0.0581 (0.0141)	0.0001*	0.4762	0.2268	1.4851 (2.2203)
Equation: RNDZ = 0.0581(H) + 1.4851					
Humeral length in cm	0.1476 (0.0597)	0.0164*	0.3089	0.0954	6.1698 (1.8084)
Equation: RNDZ = 0.1476(L) + 6.1698					

*Significant at p < 0.05.

*Abbreviation* - A: age in years, F: female (input is “1” if the particular cadaver is female, “0” if male), H: height in centimetres (cm), L: humeral length in cm, M: male (input is “1” if the particular cadaver is male, “0” if female), RNDZ: radial nerve danger zone in cm, SE: standard error

Multivariate linear regression analyses with the RNDZ as the dependent variable showed that combining height and at least one other variable incrementally increased the predictive power of the model compared to height alone in the whole sample (Table III). While considering all other variables (height, humeral length, age and sex) as predictors accounts for the highest proportion of variance in the danger zone (R^2^ = 29.55%), a minimal decline in this metric was observed when the humeral length was not considered (R^2^ = 29.47%). Sex-disaggregated analyses revealed that all model sets predicted the RNDZ in females with statistical significance and R^2^ > 57%. On the other hand, only multivariate models that included height and age were statistically significant predictors of RNDZ in males, albeit accounting for its variability to a lesser extent than those for females (R^2^ < 22.00%).

**Table III:** Multivariate regression model building results with danger zone as the dependent variable

Predictor Model Set	Equation	p-value	R^2^
Female Subset of Sample			
Height, age	RNDZ = 0.0869(H) – 0.0022(A) – 2.5251	0.0001*	0.5795
Height, humeral length	RNDZ = 0.0847(H) – 0.0179(L) – 2.8296	0.0001*	0.5790
Height, humeral length, age	RNDZ = 0.0554(H) + 0.0089(L) – 0.0101(A) + 2.1738	0.0004*	0.5858
Male Subset of Sample			
Height, age	RNDZ = 0.0821(H) – 0.0248(A) – 1.3032	0.0192*	0.2129
Height, humeral length	RNDZ = 0.0737(H) – 0.0335(L) – 0.1334	0.0872	0.1374
Height, humeral length, age	RNDZ = 0.0950(H) – 0.0682(L) – 0.0260(A) – 1.2204	0.0446*	0.2200
Full Aggregate of Sample			
Height, age	RNDZ = 0.0569(H) – 0.0099(A) + 2.1872	0.0003*	0.2478
Height, sex	RNDZ = 0.0765(H) – 0.5392(S) – 0.5468	0.0002*	0.2591
Height, humeral length	RNDZ = 0.0600(H) – 0.0113(L) + 1.5198	0.0006*	0.2271
Height, age, sex	RNDZ = 0.0793(H) – 0.0132(A) – 0.6659(S) – 0.0872	0.0002*	0.2947
Height, humeral length, age	RNDZ = 0.0554(H) + 0.0089(L) – 0.0101(A) + 2.1738	0.0011*	0.2480
Height, humeral length, sex	RNDZ = 0.0780(H) – 0.0089(L) – 0.5384(S) – 0.5163	0.0007*	0.2593
Height, humeral length, age, sex	RNDZ = 0.0762(H) + 0.0189(L) – 0.0137(A) – 0.6719(S) – 0.1366	0.0006*	0.2955

*Significant at p < 0.05.

*Abbreviation* - A: age in years, H: height in centimetres (cm), L: humeral length in cm, RNDZ: radial nerve danger zone in cm, S: sex (0 = female, 1 = male), SE: standard error

## Discussion

In this study of Filipino formalin-preserved cadavers, we estimated the mean RNDZ, measured from the lateral epicondyle to the exit point of the nerve in the lateral intermuscular septum, to be 10.6cm (95% CI: 10.3cm, 10.9cm). This estimate is comparable with the estimate from investigations in Taiwan^7^ and Ireland^9^ while being statistically smaller than those from Italy^12^ (Table IV). However, compared to data from the United States, our estimate is similar to that of only one^13^ and is statistically smaller than those from the remaining four studies^14-17^. This raises the possibility of the RNDZ being truly smaller in Filipino adult patients compared to other nationalities (especially in most non-Asian ones), and this has contextual implications for the conduct of relevant surgical interventions in this population. While we also noted that female cadavers in our study had statistically shorter height and humeral length than their male counterparts, this may likely be ungeneralisable and influenced by the minuteness of the sample size relative to the Filipino population. The RNDZ between the sexes, however, were observed to be similar.

**Table IV:** Estimates of the radial nerve danger zone, measured from the lateral epicondyle to the lateral intermuscular septum, as reported in the literature

Study	Country	n	Measured RNDZ Mean ± SD (cm)	Statistical Rank*
Gerwin *et al* (1996)^14^	USA	10	14.2 ± 0.6	1
Guse and Ostrum (1995)^15^	USA	24	12.6 ± 1.1	2
Bono *et al* (2000)^16^	USA	50	12.3 ± 2.3	2
Artico *et al* (2009)^12^	Italy	30	12.1 ± 1.3	2
Simone *et al* (2009)^17^	USA	10	12.2 ± 1.0	3
Carlan *et al* (2007)^13^	USA	27	10.9 ± 1.5	4
This study	Philippines	60	10.6 ± 1.1	4
Chou *et al* (2008)^7^	Taiwan	120	10.4 ± 2.5	4
Fleming *et al* (2004)^9^	Ireland	20	10.2 ± 0.8	4

*Determined by running the estimates in one-way analysis of variance followed by Tukey-Kramer post-hoc test; lower rank indicates statistically longer estimate and studies with the same rank indicate lack of statistically significant difference between their estimates, both at p < 0.05.

*Abbreviations* - cm: centimetre, n: number of upper extremities considered, RNDZ: radial nerve danger zone, SD: standard deviation, USA: United States of America

Furthermore, we have demonstrated that this anatomic parameter can be predicted to a certain extent using routinely collected patient information and easily obtainable intraoperative measurements. However, (1) it remains to be seen whether this would have relevant use in the future and (2) a larger sample and/or variable list may be needed to generate valid and reliable predictive models to this end. Regardless of the uncertainty on the value of these predictive models, an interesting secondary finding was the propensity of the variability in male RNDZ estimates to be less accounted for by the designated independent variables, individually and in combinations, than that in females.

With the increasing appeal of surgical management for humeral shaft fractures, either to allow early mobilisation or for temporary fixation, a proper understanding of the anatomic location of the radial nerve is crucial to avoid iatrogenic injury^4^. External fixation involving the humerus should be made cautiously, especially regarding inserting percutaneous pins over the distal aspect. Several studies, mostly case reports and series, highlight the occurrence of radial nerve palsy after percutaneous application of external fixator^18-23^. For open reduction and internal fixation cases using plate and screws (either the standard open or the minimally invasive plate osteosynthesis [MIPO] technique), nerve injury may occur when the plate is placed over or under the nerve and due to the surgical approach used (lateral or posterior). Insertion of intramedullary nails for humeral shaft fractures may also put the radial nerve at risk as placing the interlocking screw over the distal portion from lateral to medial may inadvertently damage this tissue^8^. In a recent survey of 151 patients with humeral shaft fracture who underwent internal fixation through plate osteosynthesis or intramedullar nailing, 9 (6%) were noted to have sustained secondary radial nerve palsy; 4 from open plate osteosynthesis, 3 from MIPO, 1 from intramedullary nailing and 1 from percutaneous external fixator application^24^. Removal of implants, especially with scarred tissue, will incur less risk if we can predict pre-operatively the radial nerve and its orientation, position and location in the distal humerus^9^. All in all, a well-informed and careful estimation of the positioning of the radial nerve, especially with regards to its anatomic relations, is paramount in ensuring a surgery with minimised adverse outcomes.

We recommend the conduct of further local cadaveric studies of similar objectives to determine whether the results of this study are replicable. Furthermore, intraoperative investigations involving consenting live patients undergoing surgery for related diseases (in the anatomic area of concern) should also be considered; such a strategy may remove the potential extraneous influence of biological death and cadaver preservation in RNDZ ascertainment. Finally, other relevant anatomical-surgical parameters other than the danger zone may also be investigated, and endeavours that would permit better RNDZ prediction (preferably using conveniently obtained parameters) are strongly encouraged.

## Conclusion

This cadaveric study provides, for the first time, an estimate of the RNDZ in Filipino adults. Understanding the possible location of the radial nerve over the distal humerus, with blunt dissection and proper visualisation of the nerve, will aid the orthopaedic surgeon in avoiding iatrogenic radial nerve palsy during patient management.
